# Motor Performance in Autistic Youth From Childhood Through Adolescence: Evidence for Both Sustained and Widening Group Differences

**DOI:** 10.1002/aur.70211

**Published:** 2026-03-05

**Authors:** Allison R. Block, Emily C. Skaletski, Claire M. Sheedy, Ella A. Vanderpool, Brittany G. Travers

**Affiliations:** ^1^ Occupational Therapy Program in the Department of Kinesiology University of Wisconsin‐Madison Madison Wisconsin USA; ^2^ Waisman Center, University of Wisconsin‐Madison Madison Wisconsin USA; ^3^ Northwestern University Feinberg School of Medicine, Institute for Public Health and Medicine, Center for Education in Health Sciences Chicago Illinois USA

**Keywords:** adolescent, autism spectrum disorder, child, hand strength, motor skills, psychomotor performance

## Abstract

Although motor‐skill differences in autistic individuals are well established, there is diverging evidence regarding what happens to motor skills in autistic children as they become adolescents. Using both cross‐sectional and longitudinal data, we examined fine and gross motor skills and grip strength of 187 autistic participants and 136 non‐autistic participants (i.e., with no known diagnoses), aged 6–18 years‐old. Participants completed the Bruininks‐Oseretsky Test of Motor Proficiency‐Short Form, Second Edition (BOT‐2 SF), and maximal grip strength testing. Linear mixed‐effects regression analyses indicated motor‐skill differences between autistic and non‐autistic participants across this age range; however, the nature of these differences depended on the specific motor domain (i.e., strength) and measure. Specifically, grip strength and BOT‐2 SF strength subtest scores showed widening group differences with increasing age, whereas overall BOT‐2 SF scores and subtests showed sustained or narrowing group differences through adolescence. However, items on the BOT‐2 SF also demonstrated substantial ceiling effects, which may obscure later group differences between autistic and non‐autistic participants and highlight the need for measures that encompass a greater range of motor skills into adolescence. These findings have important implications for healthcare, education, and community supports that address age‐related motor differences within the autistic population.

## Lay Summary

We looked at how motor skills—like grip strength and overall motor ability—vary with age in autistic and non‐autistic children and teenagers (6–18 years old). We found that autistic youth had weaker grip strength and more difficulty with motor skills compared to non‐autistic peers, with the gap in strength widening in adolescence and the gap in overall motor ability remaining the same or narrowing. Our findings show that motor differences in autism persist through adolescence and that we should carefully consider how we measure motor skills in this age range, which could inform better support for autistic people with motor differences in healthcare, education, and community settings.

## 1 Introduction

Motor‐skill differences are highly prevalent in autistic people (Bhat et al. 2011; Bhat 2020; Fournier et al. 2010; Licari et al. 2020), with a recent population‐based study suggesting that 71% of autistic children have a history of motor milestone delays (Pokoski et al. 2025). These motor differences present in a variety of ways (for a meta‐analysis, see Fournier et al. 2010), but often include decreased postural stability (Bojanek et al. 2020; Chen et al. 2019; Lim et al. 2017), decreased gross and fine motor control (Liu et al. 2021; Lloyd et al. 2013; Mohd Nordin et al. 2021), weaker grip strength (Abu‐Dahab et al. 2013; Ali et al. 2025; Travers et al. 2017), and slower shifts in eye gaze (Mosconi et al. 2013). Motor ability differences in autistic youth are reported in the first years of life (for meta‐analysis, see West 2019) and can be observed into early and late adulthood (Cassidy et al. 2016; Gowen et al. 2023; Linke et al. 2020; Minshew et al. 2004; Travers et al. 2017), potentially manifesting later in life as Parkinsonian‐like features (Geurts et al. 2022; Starkstein et al. 2015). Given that motor‐skill differences can impact everyday participation (Oliveira et al. 2021), adaptive behavior (Fears et al. 2022; MacDonald et al. 2013), and daily living skills (Jasmin et al. 2009; Skaletski et al. 2024; Travers et al. 2017, 2022), understanding how motor differences in autism occur across different ages is important. However, research regarding the patterns of motor differences for autistic youth from childhood into adolescence lacks consensus, particularly when compared to same‐aged people without a diagnosis of autism or other neurodevelopmental condition (henceforth referred to as “non‐autistic”). The purpose of this study is to understand age‐related differences in motor skills of autistic children and adolescents compared to their non‐autistic peers and what factors may be contributing to existing discrepancies in the literature.

While research in infants and toddlers suggest that motor differences between autistic and non‐autistic youth appear early and become more pronounced with age (for meta‐analysis, see West 2019), discrepancies arise regarding age‐related changes throughout late childhood and adolescence. For example, a longitudinal study by Travers et al. (2017) found a widening trajectory of group differences in grip strength and finger tapping speed between autistic and non‐autistic people aged 5–40 years, such that autistic people began to differentiate from non‐autistic people in manual motor skills around 15 years of age. These findings converge with cross‐sectional studies of grip strength (ages 5–21 years‐old; Abu‐Dahab et al. 2013) and postural stability (ages 5–52 years‐old; Minshew et al. 2004). However, recent cross‐sectional studies found that group differences in motor skills are sustained (i.e., not widening nor narrowing) as children age into adolescence and young adulthood. For example, Fears et al. (2023) found that autistic individuals demonstrated sustained group differences in postural stability as compared to non‐autistic peers aged 7–20 years‐old. Martín‐Díaz et al. (2024) also found that autistic people demonstrated sustained group differences in general motor ability compared to non‐autistic peers aged 6–18 years‐old. Therefore, evidence exists for sustained and widening group differences in motor trajectories from childhood to adolescence. Understanding the nature of age‐related differences in motor skills has important public health implications for intervention practices during adolescence and beyond, and further research is needed to determine reasons for apparent inconsistencies in the literature.

The studies that have differed in their findings of sustained (Fears et al. 2023; Martín‐Díaz et al. 2024) or diverging (Abu‐Dahab et al. 2013; Minshew et al. 2004; Travers et al. 2017) group differences in motor skills have differed in key ways. Indeed, all of the aforementioned studies used different but commonly used measures in clinical practice and research (i.e., maximal grip strength, the Bruininks‐Oseretksy Test of Motor Proficiency [BOT‐2] (Bruininks and Bruininks 2005), and postural stability measures). Each of these measures assesses different aspects of motor skills and has differing psychometric properties. Specifically, maximal grip strength testing has been done in individuals 4–90 years of age, but a large study of grip strength norms (Dodds et al. 2016) shows a smaller range of scores in the youngest children, potentially related to floor effects in this youngest cohort. Conversely, the BOT‐2, while widely used in occupational and physical therapy for individuals ages 4–21 years‐old, has been shown to have ceiling effects on individual items in non‐autistic individuals (Bardid et al. 2019; Brahler et al. 2012). These studies did not explicitly include autistic participants, making it unclear how floor or ceiling effects might impact group differences when comparing autistic and non‐autistic individuals. Therefore, the psychometric properties of these varying measures is one factor that could produce different trajectories of age‐related changes, which should be tested using different motor measures in the same sample of participants.

Considering these key gaps, the first aim of the present study was to examine age‐related differences in motor skills in autistic versus non‐autistic youth (ages 6–18 years) using common research and clinical measures, including the BOT‐2 Short Form (BOT‐2 SF) (Aim 1a) and maximal grip strength scores (Aim 1b). The second aim of this study was to investigate potential reasons for variation in group differences across this age range, including differing sample characteristics (Aim 2a), motor measure overlap (Aim 2b), measurement psychometric properties, such as ceiling/floor effects (Aim 2c), and BOT‐2 SF subtest‐level age‐related changes (Aim 2d).

## 2 Methods

### 2.1 Design

**FIGURE 1:**
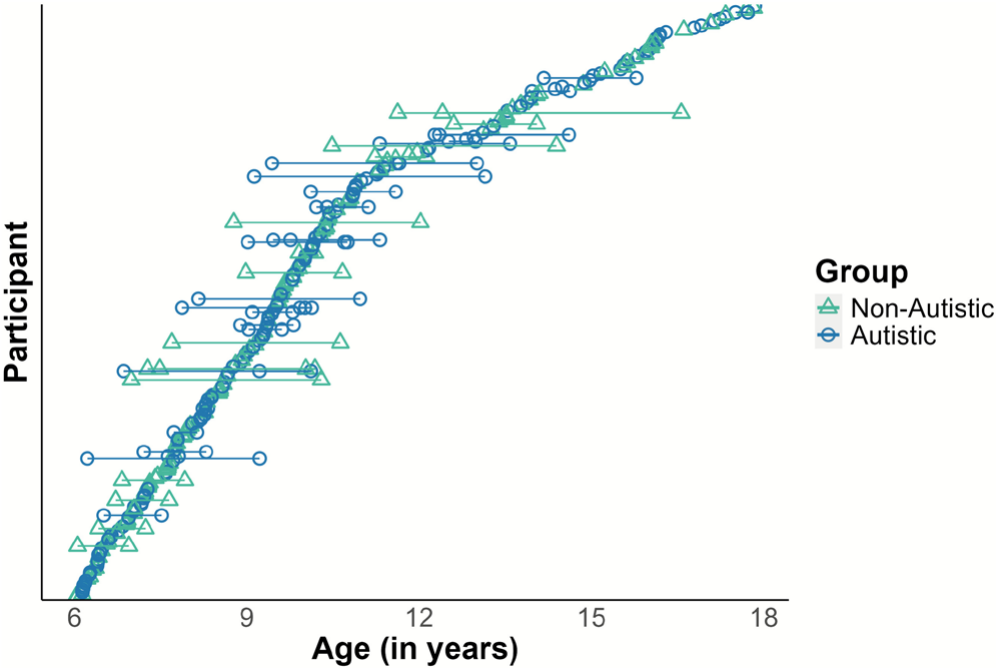
Distribution of sample by age and diagnostic group. Multiple observations/time points noted for individuals with repeated‐measures or longitudinal data.

This primarily cross‐sectional, retrospective study combined data from six previous studies (see Table S1 for more detail on each of the studies). Each study varied in their aims, although all studies were interested in motor skills in autism. Data collection for the projects occurred between 2014 and 2024. Data collection was paused in 2020 due to COVID‐19 restrictions. Participants completed the BOT‐2 SF in five of the six studies and an assessment of maximal grip strength across all six studies. As can be seen in Figure 1's depiction of participant ages, repeated observations were available in a subset of participants (*n*_autistic_ = 35, *n*_non‐autistic_ = 19), and measurements for longitudinal participants were collected 2 months to 4 years apart. The inclusion of all available data yields a greater age span for analyses and provides a more comprehensive estimate of age‐related changes in motor skills in autistic versus non‐autistic people.

### 2.2 Participants

Participants included 187 autistic and 136 non‐autistic individuals (see Table 1 for demographic information). Autistic participants were required to have a prior clinical diagnosis of autism and meet cutoff criteria on either the Autism Diagnostic Observation Scale, second edition (ADOS‐2) (Lord et al. 2012) or on the Autism Diagnostic Interview‐Revised (ADI‐R) (Rutter, Bailey, and Lord 2003; Rutter, Le Couteur, and Lord 2003), depending on the study. Non‐autistic individuals were excluded if they met cutoff criteria for autism spectrum on the ADOS‐2 (one study) or if they had elevated scores on the social communication questionnaire (SCQ) (Rutter, Bailey, and Lord 2003; Rutter, Le Couteur, and Lord 2003) (≥ 11 in one study and ≥ 15 in four studies). Children with tuberous sclerosis, Down syndrome, fragile X, or with a history of severe head injury were excluded from the study (see Table S1 for additional inclusion and exclusion criteria across the combined studies). A subset of participants with both BOT‐2 SF and maximal grip strength scores was utilized to investigate age‐related changes in motor skills in identical samples of participants for Aim 2a (see Table S2 for sample sizes for each Aim). All projects from which data were collated were approved by the University of Wisconsin‐Madison Institutional Review Board (#2014‐1499, 2018‐1067, 2014‐1248, 2016‐0785, 2016‐0441, 2016‐0784), as was the ability to combine data from these projects for our analyses (#2022‐0413). All guardians provided consent, and all youth provided assent prior to participation.

**TABLE 1:** Demographic characteristics and descriptive statistics.

	Autistic (*n* = 187)	Non‐autistic (*n* = 136)	Group difference
	*M* (SD), range OR %	*M* (SD), range OR %	*p*
Age (in years)	10.32 (3.06), 6.14–17.85	10.08 (3.07), 6.02–17.83	0.49
Sex (% female)	13.90%	31.62%	< 0.001
Participants with longitudinal data (% of total sample)			
One time point	81.28%	86.03%	
Two time points	12.83%	11.76%	
Three time points	4.28%	1.47%	
Four time points	1.60%	0.74%	
Race (% of total sample)			
American Indian/Alaskan Native	0.53%	0.00%	
Asian	4.81%	2.21%	
Black	6.42%	4.41%	
White	82.35%	77.21%	
Other/More than one	5.88%	16.18%	
Hispanic (% yes)	9.09%	5.88%	0.27
IQ	100.25 (18.78), 47–147	116.32 (13.09), 73–145	< 0.001
SRS‐2	94.48 (26.90), 24–168	19.65 (12.18), 1–58	< 0.001
Grip strength percentile			
< 10	27.27%	13.97%	
10–25	13.90%	13.97%	
25–50	9.63%	13.97%	
50–75	18.18%	19.12%	
75–90	9.63%	16.91%	
> 90	16.58%	20.44%	

Abbreviations: IQ = intelligence quotient (based on Wechsler Abbreviated Scales of Intelligence – second edition or Kaufman Brief Intelligence Test, 2nd edition); SRS‐2 = Social Responsiveness Scale, second edition.

### 2.3 Measures

#### 2.3.1 Bruininks‐Oseretsky Test of Motor Proficiency, Second Edition Short Form (BOT‐2 SF) (Bruininks and Bruininks 2005)

The BOT‐2 is a performance‐based measure that asks children to complete several fine and gross motor activities, such as hopping, drawing, and sit‐ups. Assessment norms are established for children aged 4–21 years, and the assessment is intended for practitioner and researcher use (Deitz et al. 2007 Griffiths et al. 2018). Because BOT‐2 Full Form data was not available across all studies, we used data from an abbreviated version, the BOT‐2 Short Form. Total BOT‐2 SF point scores were used for the analysis, but follow‐up tests using standard scores were performed to confirm consistency of results. To obtain BOT‐2 SF total point scores, we implemented BOT‐2 manual guidelines. Specifically, the point score total sums the “points” of each short‐form item, which is generated by the raw score (i.e., a raw score of 20 on the transferring‐pennies item is a point score of 9). For analysis of BOT‐2 SF individual‐item ceiling and floor effects, raw scores of individual items on the BOT‐2 SF were used. For BOT‐2 SF items that included more than one trial, the trial with the higher score was used for analysis. For analyses of BOT‐2 SF subtests, raw scores on the 1–2 individual BOT‐2 SF items within that subtest were *z*‐scored and averaged to generate an average *z*‐score for each subtest. Using *z*‐scored raw scores allows for greater item variability in performance for analysis. Higher BOT‐2 SF total point scores, item‐level raw scores, and average *z*‐scores are indicative of better motor ability.

#### 2.3.2 Grip Strength

Grip strength data were collected with a Jamar hand dynamometer in units of kilograms and according to the Halstead‐Reitan Battery guidelines (Heaton et al. 1991). Participants were instructed to keep their arms at their sides with their elbows at 90 degrees and squeeze the stirrup as tightly as possible. Raw maximal grip strength scores were used for analysis of grip strength trajectories, but Figure S1 illustrates the established age‐ and sex‐based population norms for grip strength (Dodds et al. 2016) superimposed on the raw grip strength data of the study. This figure demonstrates the high degree of conformity that the non‐autistic data have with the population norms.

### 2.4 Data Analysis

Analyses were conducted in R (version 4.4.1) (R Core Team 2024) with lme4 (Bates et al. 2015) and lmerTest packages (Kuznetsova et al. 2017). Graphs were made utilizing ggplot2 (Wickham 2016). All independent variables were mean‐centered prior to analysis to avoid multicollinearity, and alpha was set at *p* < 0.05. To investigate age‐related differences in motor‐skills in autistic versus non‐autistic youth, linear mixed‐effects regression (LMER) analyses of BOT‐2 SF total point scores (Aim 1a) and maximal grip strength scores (Aim 1b) as a function of age and diagnostic group were utilized. To determine statistical models of best fit for BOT‐2 and grip strength, we utilized AIC (Akaike 1973) to evaluate appropriateness of including IQ and sex as covariates due to research that indicates IQ contributes to motor ability differences (Ramos‐Sánchez et al. 2022; Surgent et al. 2021; Yu et al. 2018) and sex differences are present in grip strength (Nuzzo 2025). AIC suggested that BOT‐2 SF was best modeled quadratically with IQ as a covariate (BOT‐2 SF~Age^2 × Group + IQ + 1|participant), while grip strength was best modeled quadratically with both IQ and sex as covariates (Grip~Age^2 × Group + IQ + Sex + 1|participant). We additionally considered non‐linear spline modeling of these data, but a graph‐based visualization of the spline and quadratic smooth lines were nearly identical (Figure S2). Thus, we chose to implement linear modeling with the quadratic term to enhance parsimony of the statistical approach. Because BOT‐2 standard scores (accounting for age and sex) are typically used over total scores in clinical practice, we conducted follow‐up analyses using the same model with BOT‐2 SF standard scores.

We also investigated how sample characteristics, measurement overlap, measurement properties, and varying motor measure constructs may be contributing to discrepancies regarding age‐related changes in motor skills in autistic youth. To replicate BOT‐2 SF and grip strength findings in an identical sample of participants (Aim 2a), we used the same LMER models to investigate a subset of participants who had both BOT‐2 SF total scores and grip strength data. To evaluate how the two motor measurements differ or assess similar motor‐skill constructs (Aim 2b), Pearson's r correlations were conducted between BOT‐2 SF total point scores and maximal grip strength. Additional Pearson's r correlations were conducted between average z‐scores on the strength subtest of the BOT‐2 SF and maximal grip strength, given the potential convergence of strength constructs between these two measures. Because we observed positive skewing in maximal grip strength scores, we applied a square root transformation of grip strength across correlations. To investigate motor measurement properties, including ceiling and floor effects (Aim 2c), we investigated the percent of sample at ceiling/floor for raw scores of each item of the BOT‐2 SF, for BOT‐2 SF total point scores, and for maximal grip strength scores, both across diagnostic groups and by age. These values were examined and categorized as having substantial ceiling or floor effects if more than 15% of the sample was at the maximum or minimum score possible (Terwee et al. 2007). The number of BOT‐2 SF items that met this threshold for ceiling or floor effects by diagnostic group and age was also investigated. To better understand how subtests of the BOT‐SF reflect age‐related group differences in BOT‐2 SF total scores, we analyzed age‐related changes for each subtest of the BOT‐2 SF (Aim 2d) using the same model as Aim 1a analysis of total point scores.

## 3 Results

### 3.1 Aim 1: Age‐Related Changes in Motor‐Skills in Autistic Versus Non‐Autistic Youth

**FIGURE 2:**
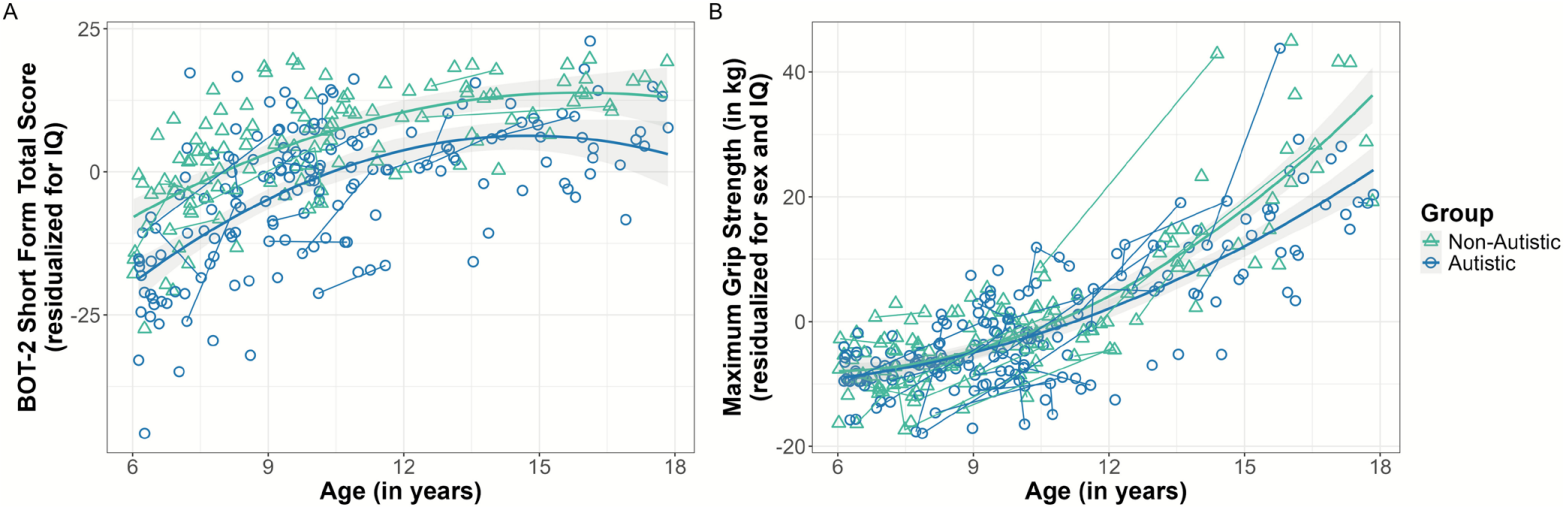
Age‐related group differences on the BOT‐2 SF (A) and maximal grip (B) in autistic people versus non‐autistic people, controlling for IQ (BOT‐2 SF and grip) and sex (grip only). BOT‐2 SF = Bruininks‐Oseretsky Test of Motor Proficiency, Second Edition, Short Form.

As can be seen in Table 2 and Figure 2A, we did not find a significant interaction effect between age and diagnostic group on BOT‐2 SF total point scores, *b* = 0.20, SE = 0.16, *p* = 0.22, *d* = 0.15 (Aim 1a). Instead, we observed significant main effects of age, group, and IQ on BOT‐2 SF scores such that non‐autistic people demonstrated higher BOT‐2 SF scores than autistic people across this age range. Analyses of BOT‐2 standard scores revealed slightly more robust group differences and an identical interaction effect size, *b* = 0.17, SE = 0.14, *p* = 0.22, *d* = 0.15 (see Table S3).

**TABLE 2:** Results of linear mixed effects analyses of age‐related motor skills in autistic versus non‐autistic people.

Variable	Term	df	SE	*t*	*p*	*d*
BOT‐2 SF	Age	279.51	0.20	14.14	< 0.001	1.69
	Age^2^	271.28	0.05	−6.26	< 0.001	−0.76
	IQ	279.26	0.03	8.18	< 0.001	0.98
	Group	255.59	0.58	−8.86	< 0.001	−1.11
	Age × Group	273.63	0.16	1.22	0.22	0.15
Grip	Age	301.61	0.14	18.09	< 0.001	2.08
	Age^2^	303.55	0.04	6.50	< 0.001	0.75
	IQ	293.21	0.02	2.91	0.01	0.34
	Sex	275.92	0.45	2.99	0.01	0.36
	Group	272.98	0.41	−2.53	0.01	−0.31
	Age × Group	297.77	0.11	−3.66	< 0.001	−0.42

*Note:* BOT‐2 SF = Bruininks‐Oseretsky Test of Motor Proficiency, Second Edition, Short Form; IQ = intelligence quotient.

As can be seen in Table 2 and Figure 2B, we found a small‐sized, significant interaction effect between age and diagnostic group on grip strength scores, with a widening of group differences in grip strength as age increased among participants, *b* = −0.42, SE = 0.11, *p* = < 0.001, *d* = −0.42 (Aim 1b). Visual examination of Figure 2B suggests that group differences in grip strength may emerge around the age of 13. To confirm this, independent sample *t*‐tests were conducted in participants under the age of 13 years and in those over the age of 13 years. These *t*‐tests supported that significant group differences occurred at approximately 13 years of age. Autistic participants below 13 years old demonstrated slightly weaker grip strength (*M* = 13.68, SD = 6.54) than non‐autistic participants below 13 years old (*M* = 14.28, SD = 5.00), but this difference was not statistically significant and had a small effect size, *t*(243) = 0.82, *p* = 0.41, *d* = 0.11. In contrast, autistic participants above 13 years of age demonstrated weaker grip strength (*M* = 32.59, SD = 10.35) than non‐autistic participants above 13 years old (*M* = 39.54, SD = 13.16), and this difference was statistically significant with a medium effect size, *t*(65) = 2.32, *p* = 0.02, *d* = 0.66.

### 3.2 Aim 2: Investigate Reasons for Discrepancies Regarding Changes in Motor‐Skills in Autistic Youth With Age

Analyses within identical samples of only participants with both BOT‐2 SF total point scores and grip strength data revealed nearly identical group‐level patterns and main effects as the results with the entire sample (Aim 2a) (see Table S4 and Figure S3).

Correlations between BOT‐2 SF total point scores and maximal grip strength indicated a moderate‐sized correlation between the two measures, *r* = 0.55, *p* < 0.001 (see Figure S4), with diminished but significant relations found after controlling for age, *r* = 0.30, *p* < 0.001 (Aim 2b). Correlations between average *z*‐scores of the BOT‐2 SF strength subtest and maximal grip strength also indicated a moderate‐sized correlation, *r* = 0.50, *p* < 0.001, with diminished but significant relations found after controlling for age, *r* = 0.35, *p* < 0.001.

**FIGURE 3:**
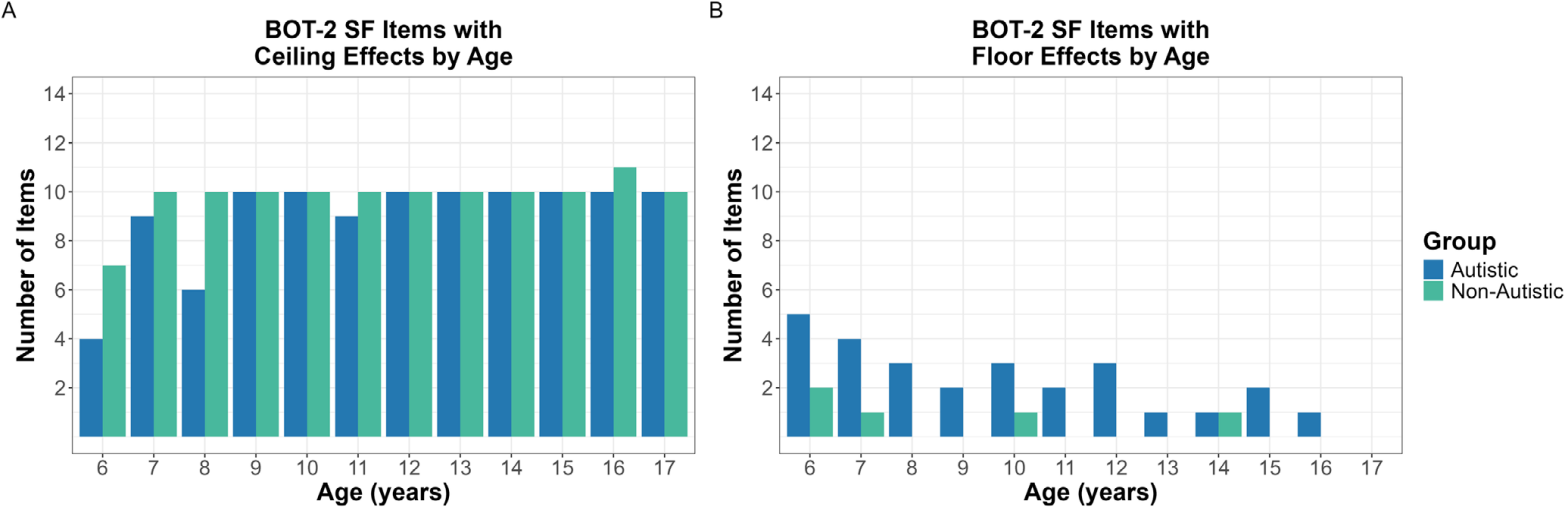
Number of BOT‐2 SF items (of 14 total) that demonstrate (A) ceiling effects or (B) floor effects within each age group in autistic and non‐autistic participants. BOT‐2 SF items were classified as having a ceiling or floor effect if more than 15% of participants within that age group were at ceiling or floor, respectively (Terwee et al. 2007).

As shown in Table 3, 11 of 14 individual BOT‐2 SF items demonstrated ceiling or floor effects, while no ceiling or floor effects were observed on BOT‐2 SF total point scores or maximal grip strength (Aim 2c). Two of these BOT‐2 SF items—drawing a star shape and jumping—demonstrated both ceiling and floor effects, and one of these BOT‐2 SF items demonstrated only a floor effect (push‐ups). Further, as shown in Figure 3, starting at age 7 and persisting through age 17, ceiling effects were present in at least 10 of 14 (71.43%) BOT‐2 SF items for the non‐autistic group. In the autistic group, ceiling effects on 10 of 14 BOT‐2 SF items emerged at age 9 and were relatively consistent through age 17. Conversely, floor effects on the BOT‐2 SF were most common in younger autistic individuals (evident in 5 of 14 BOT‐2 SF items in autistic participants at the age of 6). For the proportion of participants at ceiling/floor with age for each BOT‐2 SF item in autistic versus non‐autistic participants, see Figure S5.

**TABLE 3:** Percent of sample at ceiling or floor for each item on the BOT‐2 SF, for BOT‐2 SF total scores, and for maximal grip strength in each diagnostic group (autistic and non‐autistic).

Variable	Percent of sample at ceiling		Percent of sample at floor	
	Autistic	Non‐autistic	Autistic	Non‐autistic
Drawing	***46.99%***	***65.83%***	0.60%	0.00%
Folding	***34.34%***	***58.33%***	5.42%	1.67%
Shapes: Square	***85.54%***	***92.50%***	1.20%	0.83%
Shapes: Star	***32.53%***	***63.33%***	***19.28%***	4.16%
Jumping	***58.43%***	***87.50%***	***17.47%***	1.67%
Tapping	***82.53%***	***99.16%***	3.61%	0.00%
Walking	***89.76%***	***98.33%***	1.81%	0.00%
Standing	***40.96%***	***72.50%***	1.81%	0.00%
Catching	***58.43%***	***62.50%***	4.22%	1.67%
Dribbling	***22.89%***	***44.16%***	4.82%	1.67%
Pushups	0.00%	0.00%	***46.99%***	14.16%
Pennies	0.60%	0.00%	0.60%	0.00%
Hopping	0.60%	4.16%	5.42%	0.00%
Sit‐ups	0.00%	0.00%	13.25%	0.83%
BOT‐2 SF Total	0.00%	0.00%	0.00%	0.00%
Maximal grip (kg)	0.00%	0.00%	0.00%	0.00%

*Note:* Ceiling or floor effects (above 15%) are bolded and italicized.

Abbreviations: BOT‐2 SF = Bruininks‐Oseretsky Test of Motor Proficiency, Second Edition, Short Form; kg = kilograms.

**FIGURE 4:**
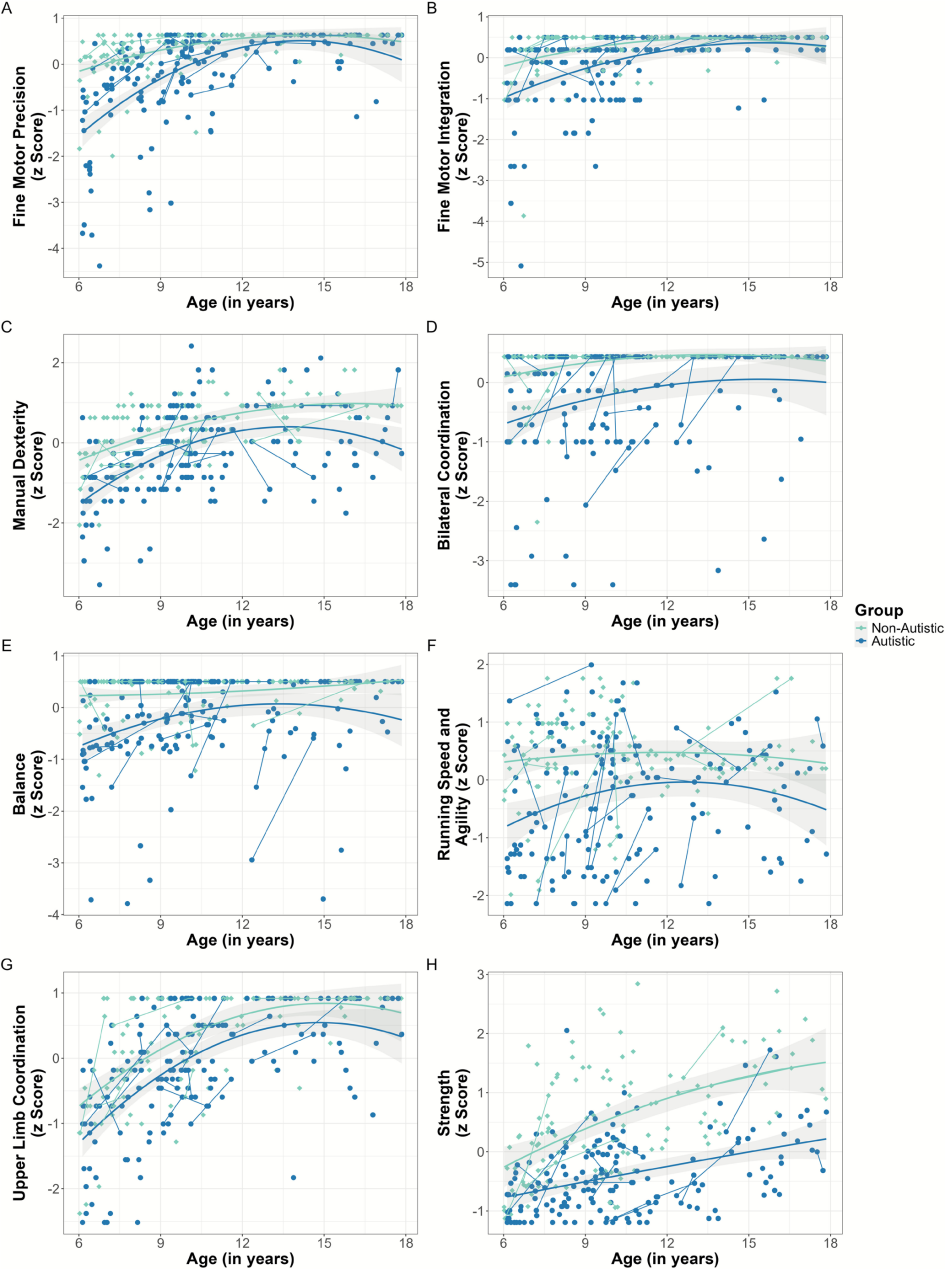
Age‐related group differences on each individual subtest of the BOT‐2 SF in autistic versus non‐autistic people, controlling for IQ. Autistic and non‐autistic youth display significantly different age‐related changes in fine motor precision (A), fine motor integration (B), and strength (H) subtests. Group differences were maintained with age on manual dexterity (C), bilateral coordination (D), balance (E), running speed and agility (F), and upper limb coordination (G) subtests. BOT‐2 SF = Bruininks‐Oseretsky Test of Motor Proficiency‐Short Form, Second Edition.

As can be seen in Table 4 and Figure 4, we found a small‐to‐medium‐sized interaction effect between age and diagnostic group on three subtests of the BOT‐2 SF, with narrowing group differences in fine motor precision and fine motor integration subtests and widening group differences on the strength subtest with age (Aim 2d). The five remaining subtests of the BOT‐2 SF showed significant main effects of group but no age‐by‐group interactions. However, the high number of participants at ceiling for fine motor integration, fine motor precision, bilateral coordination, balance, and upper limb coordination subtests is evident in Figure 3 and may impact the interpretability of these results. Moreover, less substantial, but still present, floor effects were observed in the strength subtests, particularly in the autistic participants. Main effects of age, group, and IQ on BOT‐2 SF subtests are also presented in Table 4.

**TABLE 4:** Main and interaction effects between age and diagnostic group (autistic vs. non‐autistic) on average *z*‐scores on each subtest of the BOT‐2 SF.

BOT‐2 SF subtest	Effect	SE	*t*	*p*	*d*
Fine motor precision (Drawing*, Folding*)	Age	0.01	11.40	< 0.001	1.37
	Age^2^	0.01	−6.08	< 0.001	−0.73
	Group	0.04	−3.76	< 0.001	−0.48
	IQ	0.01	9.01	< 0.001	1.08
	Age × Group	0.01	4.13	< 0.001	0.50
Fine motor integration (Copy square*, Copy star*)	Age	0.02	6.99	< 0.001	0.84
	Age^2^	0.01	−2.89	0.01	−0.35
	Group	0.05	−3.07	0.01	−0.39
	IQ	0.01	4.47	< 0.001	0.54
	Age × Group	0.01	2.47	0.01	0.30
Manual dexterity (Pennies)	Age	0.02	10.42	< 0.001	1.27
	Age^2^	0.01	−5.52	< 0.001	−0.66
	Group	0.05	−4.17	< 0.001	−0.53
	IQ	0.01	6.55	< 0.001	0.79
	Age × Group	0.01	0.14	0.89	0.02
Bilateral coordination (Jumping*, Tapping*)	Age	0.02	3.85	< 0.001	0.47
	Age^2^	0.01	−1.92	0.06	−0.23
	Group	0.05	−4.19	< 0.001	−0.54
	IQ	0.01	4.23	< 0.001	0.51
	Age × Group	0.01	1.28	0.20	0.16
Balance (Walking*, Standing*)	Age	0.02	3.43	< 0.001	0.42
	Age^2^	0.01	−1.73	0.08	−0.21
	Group	0.05	−3.78	< 0.001	−0.48
	IQ	0.01	4.58	< 0.001	0.56
	Age × Group	0.01	1.02	0.31	0.13
Running speed and agility (Hopping*)	Age	0.02	2.53	0.01	0.31
	Age^2^	0.01	−2.60	0.01	−0.31
	Group	0.06	−4.94	< 0.001	−0.61
	IQ	0.01	2.99	0.01	0.36
	Age × Group	0.02	0.93	0.35	0.11
Upper‐limb coordination (Catching*, Dribbling*)	Age	0.02	12.79	< 0.001	1.54
	Age^2^	0.01	−5.77	< 0.001	−0.69
	Group	0.05	−3.52	< 0.001	−0.44
	IQ	0.01	2.03	0.04	0.24
	Age × Group	0.01	0.73	0.47	0.09
Strength (Push‐ups*, Sit‐ups)	Age	0.02	8.26	< 0.001	0.99
	Age^2^	0.01	−1.25	0.21	−0.15
	Group	0.05	−9.03	< 0.001	−1.14
	IQ	0.01	3.53	< 0.001	0.42
	Age × Group	0.01	−2.64	0.01	−0.32

*Note:* Items that contribute to each subtest are indicated in parentheses and are marked with an asterisk if impacted by ceiling or floor effects.

Abbreviations: BOT‐2 SF = Bruininks‐Oseretsky Test of Motor Proficiency, Second Edition, Short Form; IQ = intelligence quotient.

## 4 Discussion

This study sought to investigate age‐related differences in motor skills in autistic and non‐autistic youth from childhood into adolescence. We analyzed primarily cross‐sectional motor skills, obtained from the BOT‐2 SF and grip strength, in children aged 6–18 years. Our results support group differences in motor ability between autistic and non‐autistic youth, adding to a large body of literature supporting differences in motor ability in autistic individuals (Bhat et al. 2011; Bhat 2020; Fournier et al. 2010; Licari et al. 2020; Pokoski et al. 2025). Our findings also add clarification to discrepancies in the literature regarding how group differences may change as a function of age. Specifically, general motor skills (as measured by BOT‐2 SF total scores) appeared as early as 6 years old and were maintained through adolescence. However, group differences in grip strength were initially minimal but showed group differences after 13 years of age. Importantly, these results remained when examining across the same participants, suggesting that both sustained and widening group differences are possible within the same group of individuals. However, other important considerations emerged from our findings. Specifically, grip strength and the strength subtest of the BOT‐2 SF both converged to demonstrate widening group differences with age, suggesting that strength may be a domain where this widening is likely to occur throughout adolescence. Second, an examination of BOT‐SF subtests suggested patterns of widening, narrowing, and sustained group differences accompanied by substantial ceiling effects and some floor effects which likely impacted these patterns of results. Taken together, these findings have important implications for how motor abilities in autistic youth are assessed and addressed, both in clinical practice and future research, which is discussed in greater detail below.

Our findings of widening group differences in grip strength and BOT‐2 SF strength measures is consistent with previous longitudinal and cross‐sectional work (Abu‐Dahab et al. 2013; Travers et al. 2017) and suggest that strength may be a key target area for intervention in autistic youth. As in the aforementioned work, this study did not find evidence of age‐related decreases in strength within the autistic group. Instead, gaps in strength gains between autistic and non‐autistic youth increased into adolescence, which may be the result of an earlier plateau in strength gains within the autistic group beginning in adolescence (Travers et al. 2017). While this study did not observe floor effects in grip strength, moderate floor effects on the BOT‐2 SF strength subtest items were present, suggesting the need for potentially more sensitive strength measures to detect individual differences within the lower ranges of the strength scale. Therefore, it is possible that floor effects impacted this widening pattern of group differences, which will need to be investigated in future work with more low‐range‐sensitive measures. Nevertheless, decreased strength in autistic adolescents compared to age‐matched non‐autistic peers, especially if persisting into adulthood, may impact performance of daily living tasks, as grip strength has been shown to relate to both concurrent and future daily living skills (Travers et al. 2017). Moreover, physical activity interventions in autism have shown positive effects for strength with cardiovascular benefits (as reviewed in Ataíde et al. (2026)), suggesting that these types of interventions may be an important avenue to mitigate group differences in strength with age. Future research is needed to determine if autistic adults are at a greater risk for weaker hand strength and/or general muscle weakening (e.g., sarcopenia) with age, as decreased strength is associated with increased risk of falls, morbidity, and mortality in older adults (for a review see Cruz‐Jentoft and Sayer 2019).

Our results also suggest that motor measure properties may be key contributors to differing patterns of age‐related trajectories. Specifically, the high prevalence of ceiling effects and moderate floor effects encountered on individual items of the BOT‐2 SF may have hindered our ability to depict valid age‐related differences in motor skills via this measure. While previous studies have identified BOT‐2 SF ceiling effects in non‐autistic youth (Bardid et al. 2019; Brahler et al. 2012), this study is the first to report ceiling effects in both autistic and non‐autistic youth. These ceiling effects and reduced variability in performance into adolescent years, more so for non‐autistic participants, may make it appear that group differences are sustained or narrowing into adolescence, which is important for consideration in both clinical and research contexts. Indeed, fine motor integration and fine motor precision subtests were disproportionately impacted by ceiling effects and indicated narrowing group differences; thus, we believe these results should be interpreted with caution. Overall, these findings point to a need for more comprehensive measures of motor skills that can track dynamic changes in motor performance during youth into adulthood. To address this issue, the latest revision of the BOT (BOT‐3; Bruininks and Bruininks 2024) has added more challenging tasks and growth scale values to many of its items, but measures that incorporate basal and ceiling scores for tasks should also be considered. Additionally, innovative technologies such as AI‐based or digital assessments of movement show promise to distinguish motor patterns between autistic and non‐autistic individuals (Gargot et al. 2022). These new technologies may also be able to detect dynamically changing motor patterns across the lifespan within and between individuals, which will be key for future research.

The findings also have important implications for providing better support for autistic people with motor differences in healthcare, education, and community settings. Despite the current and prior findings of motor differences in autistic adolescents (Bhat et al. 2011; Bhat 2020; Fournier et al. 2010) across several motor domains (Abu‐Dahab et al. 2013; Bojanek et al. 2020; Lim et al. 2017; Mosconi et al. 2013; Travers et al. 2017), therapeutic services for autistic youth typically decline during adolescent years (Laxman et al. 2019) and demonstrate a steep drop‐off directly after high school completion (Cidav et al. 2013). Therefore, there is a clear mismatch between the availability of these services and the motor differences in autism that are sustained or even widening during this time. It is possible that ceiling effects on measures frequently used in clinical practice, like the BOT‐2, are contributing to this “age‐out” process, in which reaching ceiling on individual items gives the impression that autistic youth are catching up or making similar gains in motor ability as their non‐autistic peers. Therefore, there is a clear need for clinically feasible assessments with strong psychometric properties able to detect a wide range of motor abilities, ideally across the lifespan. In healthcare settings, assessments that longitudinally track motor differences could enhance care and potentially flag individuals across the lifespan who may benefit from earlier or ongoing intervention to decrease the likelihood of sarcopenia or fall risks with age.

In addition to continued intervention throughout this age range, environments that are more physically accessible would likely better address motor challenges in autism and increase the likelihood of meaningful participation in home, community, and educational settings. Autistic people who experience motor differences may encounter challenges completing daily tasks, such as bathing, dressing, and cleaning/organizing (Travers et al. 2022). Additional tools or environmental aids may enhance an individual's ability and self‐efficacy to complete these tasks. Further, transportation outside of the home may entail expectations for public transportation, which may entail sustaining balance while on a subway or bus, or driving, which requires a highly complex symphony of motor activity. Environments or activities that can support more motorically accessible modes of transportation may be beneficial to autistic individuals who experience more pronounced motor differences. Similarly, in school settings, this age range often experiences ever‐increasing expectations for efficient handwriting or typing to complete educational assignments or testing, which may negatively impact those with more pronounced motor differences. Accommodations within schools that address these motor differences are likely to be of benefit. Therefore, community and educational awareness of motor differences that are common in autistic youth may lead to better environmental fits which can replace or supplement interventions specifically aimed to enhance motor skills.

A strength of the present study was the inclusion of a large sample size of 323 participants ranging from 6 to 18 years of age, as a large sample size is critical for addressing a heterogeneous diagnosis such as autism. Despite our large sample size, our sample was disproportionately male (79%) and identified as White (80%), which limits the generalizability of our findings to the general population. Our inclusion of data collected over multiple observations within a subset of participants contextualizes our cross‐sectional findings, providing preliminary insight into how motor skills may change over time in autistic versus non‐autistic youth. However, the number of participants with multiple observations was limited. The present study may act as a foundation for future longitudinal research, as additional longitudinal research is needed to better understand motor‐skill development over time in autistic and non‐autistic individuals. Further, while our study added to the literature by investigating a sample of participants beyond the ages of infancy and toddlerhood, research regarding age‐related changes into mid‐ and late adulthood is still lacking and required to capture a larger picture of motor‐skill trajectories. While the present study is strengthened by its consideration of motor measure properties, ceiling and floor effects on individual items of the BOT‐2 SF were prevalent, suggesting that it may not be an appropriate measure for assessing motor skills into late childhood and adolescence, particularly for non‐autistic individuals. Furthermore, use of BOT‐2 SF subtests, which only consist of one to two items each from the full form, may have limited our ability to represent age‐related changes across domains, and future research regarding development of motor ability subcomponents is needed.

## 5 Conclusions

The present study examined the age‐related group differences in motor performance between autistic and non‐autistic youth from childhood into adolescence. Our results suggest that different motor assessments lead to differing patterns of age‐related group differences in motor skills in autistic youth compared to non‐autistic youth. The BOT‐2 SF and grip strength demonstrate distinct trajectories of age‐related group differences but converge to suggest growing group differences in strength in adolescence. Further, ceiling and floor effects on individual items of the BOT‐2 SF measure may be contributing to diverging results. Future research examining how various motor constructs change over time is required to better understand motor development of autistic youth. In addition, our results indicate a need for additional assessments that allow for better detection of motor differences into adolescence and young adulthood. Our findings affirm that motor skills differ in autistic individuals, which may impact participation and performance in everyday tasks. As such, it is imperative to investigate the influence of access and timing of interventions targeting motor performance in autistic youth.

## Funding

This research was supported by Carla & Mike Austin Faculty funds (to Brittany G. Travers), the Hartwell Foundation's Individual Biomedical Award (to Brittany G. Travers), the Brain and Behavior Research Foundation's NARSAD Young Investigator Award (to Brittany G. Travers), the University of Wisconsin‐Madison Office of the Vice Chancellor for Research's Fall Competition Award (to Brittany G. Travers), the University of Wisconsin‐Madison Office of the Vice Chancellor for Research's Spring Interdisciplinary Competition Award (to Brittany G. Travers), the Friends of the Waisman Center, and the Eunice Kennedy Shriver National Institute of Child Health and Human Development (P30 HD003352, U54 HD090256, and P50 HD105353 to the Waisman Center and R01 HD094715 and R01 HD094715‐A1 to Brittany G. Travers). The research was also supported by the Marsh Fellowship, Carns Fellowship, and Wisconsin Distinguished Graduate Fellowship at the University of Wisconsin‐Madison (to Emily C. Skaletski). The content is solely the responsibility of the authors and does not necessarily represent the official views of the National Institute of Child Health & Development nor the National Institutes of Health.

## Ethics Statement

This study was performed in line with the ethical standards of the institutional review board and the 1964 Declaration of Helsinki and later amendments. Approval was granted by the Institutional Review Board (#2014‐1499, 2018‐1067, 2014‐1248, 2016‐0785, 2016‐0441, 2022‐0413).

## Consent

Parents or legal guardians provided informed consent, and participants provided informed assent.

## Conflicts of Interest

The authors declare no conflicts of interest.

## Supporting information


**Table S1:** Additional inclusion/exclusion criteria that varied across studies.
**Table S2:** Participants included in each Aim and corresponding analysis.
**Table S3:** Results of linear mixed effects analyses of age‐related motor skills in autistic versus non‐autistic people using BOT‐2 SF standard scores over total point scores.
**Table S4:** Results of linear mixed effects analyses of age‐related motor skills in autistic versus non‐autistic youth, in the identical samples of participants for BOT‐2 SF and grip strength. Patterns of age‐related changes in this subset of participants remain nearly identical to patterns of age‐related changes in the entire sample.
**Figure S1:** Age‐related changes in maximal grip in autistic versus non‐autistic youth with percentile rank overlays. Panels A and B show participant grip strength overlaid with sex‐specific normative percentile lines (10th, 25th, 50th, 75th, and 90th percentiles) for males and females, respectively. BOT‐2 SF = Bruininks‐Oseretsky Test of Motor Proficiency, Second Edition, Short Form.
**Figure S2:** Age‐related changes on the BOT‐2 SF (first row) and maximal grip (second row) in autistic versus non‐autistic youth using linear (left column) versus spline (right column) modeling. Panels A and C show linear modeling of BOT‐2 SF and maximal grip strength scores, respectively, Panels B and D show spline modeling of BOT‐2 SF and maximal grip strength scores, respectively. Because linear (with quadratic fit) and spline modeling rendered nearly identical fit lines, we opted to report the linear modeling for parsimony.
**Figure S3:** Age‐related changes on the BOT‐2 SF (A) and maximal grip (B) in autistic versus non‐autistic people, controlling for IQ (BOT‐2 SF and grip) and sex (grip only), in identical samples of participants. Each participant in this subset had complete BOT‐2 SF and grip strength data. Patterns of age‐related changes in this subset of participants remain nearly identical to patterns of age‐related changes in the entire sample.
**Figure S4:** Correlations between maximal grip strength and BOT‐2 SF total point scores (A) and BOT‐2 SF strength subtest average *z*‐scores (B). Maximum grip strength was moderately correlated with both BOT‐2 SF total point scores and BOT‐2 SF average z‐scores on the strength subtest. BOT‐2 SF = Bruininks‐Oseretsky Test of Motor Proficiency, Second Edition, Short Form.
**Figure S5:** Proportion of participants at ceiling and floor on each item of the BOT‐2 SF by age in autistic versus non‐autistic participants. The black line represents 15% cutoff utilized for classifying ceiling/floor effects. BOT‐2 SF = Bruininks‐Oseretsky Test of Motor Proficiency, Second Edition, Short Form.

## Data Availability

The data that support the findings of this study are openly available in NIMH Data Archive at https://nda.nih.gov/study.html?id=3106, reference number 3016.
