# Supplementary material for: Motor Performance in Autistic Youth From Childhood Through Adolescence: Evidence for Both Sustained and Widening Group Differences
Source: Autism Res. 2026 Mar 5;19(4):e70211. doi: 10.1002/aur.70211 (PMC13087827; doi:10.1002/aur.70211)

Supplementary Materials for Motor Performance in Autistic Youth from Childhood through Adolescence: Evidence for both Sustained and Widening Group Differences

*Supplementary Table 1.* Additional inclusion/exclusion criteria that varied across studies.

| **Study IRB #** | **Additional Inclusion/Exclusion Criteria** | **Age range (years)** | ***n*** |
| --- | --- | --- | --- |
| 2016-0441 | Participants were excluded if they had intellectual disability (IQ<70) or contraindications to MRI. | 6-10 | 63 |
| 2018-1067 | Participants were required to be verbal with an IQ >60 and have no contraindications to MRI. Participants were also excluded if they had significant, uncorrected hearing or vision loss, as assessed through caregiver report. | 6-10 | 107 |
| 2016-0784 | Participants were excluded if they had an intellectual disability (IQ<70) or contraindications to MRI. | 13-17 | 57 |
| 2014-1499 | Participants were excluded if they had an intellectual disability (IQ<70). Non-autistic participants were excluded if they had first-degree relative with ASD. | 13-17 | 33 |
| 2016-0785 | Participants were required to have adequate vision and verbal communication skills and no prior diagnosis of intellectual disability. Non-autistic participants were excluded if they had first-degree relative with ASD. | 10-12 | 35 |
| 2014-1248 | Non-autistic participants were excluded if they had first-degree relative with ASD. | 6-17 | 28 |

*Note*. ASD = Autism spectrum disorder, MRI = Magnetic resonance imaging, IQ = intelligence quotient (based on Wechsler Abbreviated Scales of Intelligence-2nd edition or Kaufman Brief Intelligence Test, 2nd edition); SRS-2 = Social Responsiveness Scale, 2nd edition,

*Supplementary Table 2.* Participants included in each Aim and corresponding analysis.

| **Aim 1: Examine age-related changes in motor skill development in autistic vs. non-autistic youth.** | | | | | |  |
| --- | --- | --- | --- | --- | --- | --- |
|  | **Autistic (*n*)** | | **Non-Autistic (*n*)** | | |  |
| **Aim 1a: BOT-2 SF** |  | |  | | |  |
| One time point | 131 | | 102 | | |  |
| Two time points | 24 | | 15 | | |  |
| Three time points | 8 | | 2 | | |  |
| Four time points | 3 | | 1 | | |  |
| **Aim 1b: Grip** |  | |  | | |  |
| One time point | 144 | | 115 | | |  |
| Two time points | 23 | | 16 | | |  |
| Three time points | 8 | | 2 | | |  |
| Four time points | 3 | | 1 | | |  |
| **Aim 1b Follow-up: Inflection point of grip strength** | |  | |  | | |
| 6.0-13.0 years-old | | 139 | | 106 | | |
| 13.1-18.0 years-old | | 39 | | 28 | | |
| **Aim 2: Investigate reasons for discrepancies regarding motor-skill development in autistic youth over time.** | | | | | |  |
| **Aim 2a: Identical sample BOT-2 SF + Grip** |  | | | | |  |
| One time point | 124 | | 100 | | |  |
| Two time points | 23 | | 15 | | |  |
| Three time points | 8 | | 2 | | |  |
| Four time points | 3 | | 1 | | |  |
| **Aim 2b: Overlap in motor measurement** | |  | | |  |  |
| One time point | | 124 | | 100 | | |
| Two time points | | 23 | | 15 | | |
| Three time points | | 8 | | 2 | | |
| Four time points | | 3 | | 1 | | |
| **Aim 2c: Motor measure ceiling/floor effects** |  | | |  | |  |
| BOT-2 SF (Items) |  | | |  | |  |
| One time point | 131 | | 102 | | |  |
| Two time points | 24 | | 15 | | |  |
| Three time points | 8 | | 2 | | |  |
| Four time points | 3 | | 1 | | |  |
| Grip |  | |  | | |  |
| One time point | 144 | | 115 | | |  |
| Two time points | 23 | | 16 | | |  |
| Three time points | 8 | | 2 | | |  |
| Four time points | 3 | | 1 | | |  |
| **Aim 2d: BOT-2 SF subtest analyses** | |  | | |  |  |
| One time point | | 131 | | 102 | | |
| Two time points | | 24 | | 15 | | |
| Three time points | | 8 | | 2 | | |
| Four time points | | 3 | | 1 | | |

*Note*. BOT-2 SF = Bruininks-Oseretsky Test of Motor Proficiency, Second Edition, Short Form.

*Supplementary* *Table 3.* Results of linear mixed effects analyses of age-related motor skills in autistic versus non-autistic people using BOT-2 SF standard scores over total point scores.

| **Variable** | **Term** | ***df*** | ***SE*** | ***t*** | ***p*** | ***d*** |
| --- | --- | --- | --- | --- | --- | --- |
| **BOT-2 SF** (Standard scores) | **Age** | 279.96 | 0.17 | -3.03 | .01 | -0.36 |
|  | **Age^2^** | 265.68 | 0.04 | 0.52 | .60 | 0.06 |
|  | **IQ** | 279.86 | 0.02 | 7.03 | <.001 | 0.84 |
|  | **Group** | 256.97 | 0.49 | -9.56 | <.001 | -1.19 |
|  | **Age*Group** | 275.33 | 0.14 | 1.23 | .22 | 0.15 |

*Note*. BOT-2 SF = Bruininks-Oseretsky Test of Motor Proficiency, Second Edition, Short Form, IQ = intelligence quotient.

*Supplementary* *Table 4.* Results of linear mixed effects analyses of age-related motor skills in autistic versus non-autistic youth, in the identical samples of participants for BOT-2 SF and grip strength. Patterns of age-related changes in this subset of participants remain nearly identical to patterns of age-related changes in the entire sample.

| **Variable** | **Term** | ***df*** | ***SE*** | ***t*** | ***p*** | ***d*** |
| --- | --- | --- | --- | --- | --- | --- |
| **BOT-2 SF** | **Age** | 269.96 | 0.20 | 14.54 | <.001 | 1.77 |
|  | **Age^2^** | 255.40 | 0.05 | -6.74 | <.001 | -0.84 |
|  | **IQ** | 269.96 | 0.03 | 7.93 | <.001 | 0.97 |
|  | **Group** | 247.52 | 0.59 | -8.59 | <.001 | -1.09 |
|  | **Age*Group** | 265.79 | 0.16 | 1.29 | 0.20 | 0.16 |
| **Grip** | **Age** | 263.11 | 0.15 | 16.79 | <.001 | 2.07 |
|  | **Age^2^** | 266.60 | 0.04 | 5.96 | <.001 | 0.73 |
|  | **IQ** | 264.62 | 0.02 | 2.94 | .01 | 0.36 |
|  | **Sex** | 251.17 | 0.47 | 3.29 | .001 | 0.42 |
|  | **Group** | 250.04 | 0.43 | -2.24 | .03 | -0.28 |
|  | **Age*Group** | 260.04 | 0.12 | -3.25 | .001 | -0.40 |

*Note*. BOT-2 SF = Bruininks-Oseretsky Test of Motor Proficiency, Second Edition, Short Form, IQ = intelligence quotient.

*Supplementary* *Figure 1.* Age-related changes in maximal grip in autistic versus non-autistic youth with percentile rank overlays. Panels A and B show participant grip strength overlaid with sex-specific normative percentile lines (10th, 25th, 50th, 75th, and 90th percentiles) for males and females, respectively. BOT-2 SF = Bruininks-Oseretsky Test of Motor Proficiency, Second Edition, Short Form.
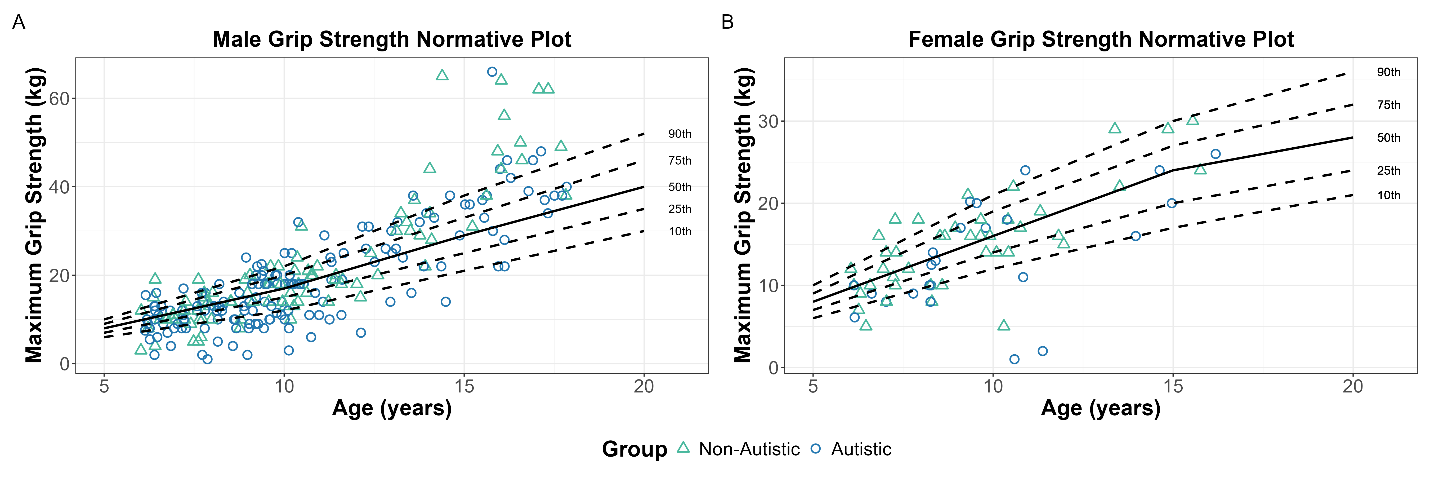


*Supplementary* *Figure 2.* Age-related changes on the BOT-2 SF (first row) and maximal grip (second row) in autistic versus non-autistic youth using linear (left column) vs. spline (right column) modeling. Panels A and C show linear modeling of BOT-2 SF and maximal grip strength scores, respectively, Panels B and D show spline modeling of BOT-2 SF and maximal grip strength scores, respectively. Because linear (with quadratic fit) and spline modeling rendered nearly identical fit lines, we opted to report the linear modeling for parsimony.


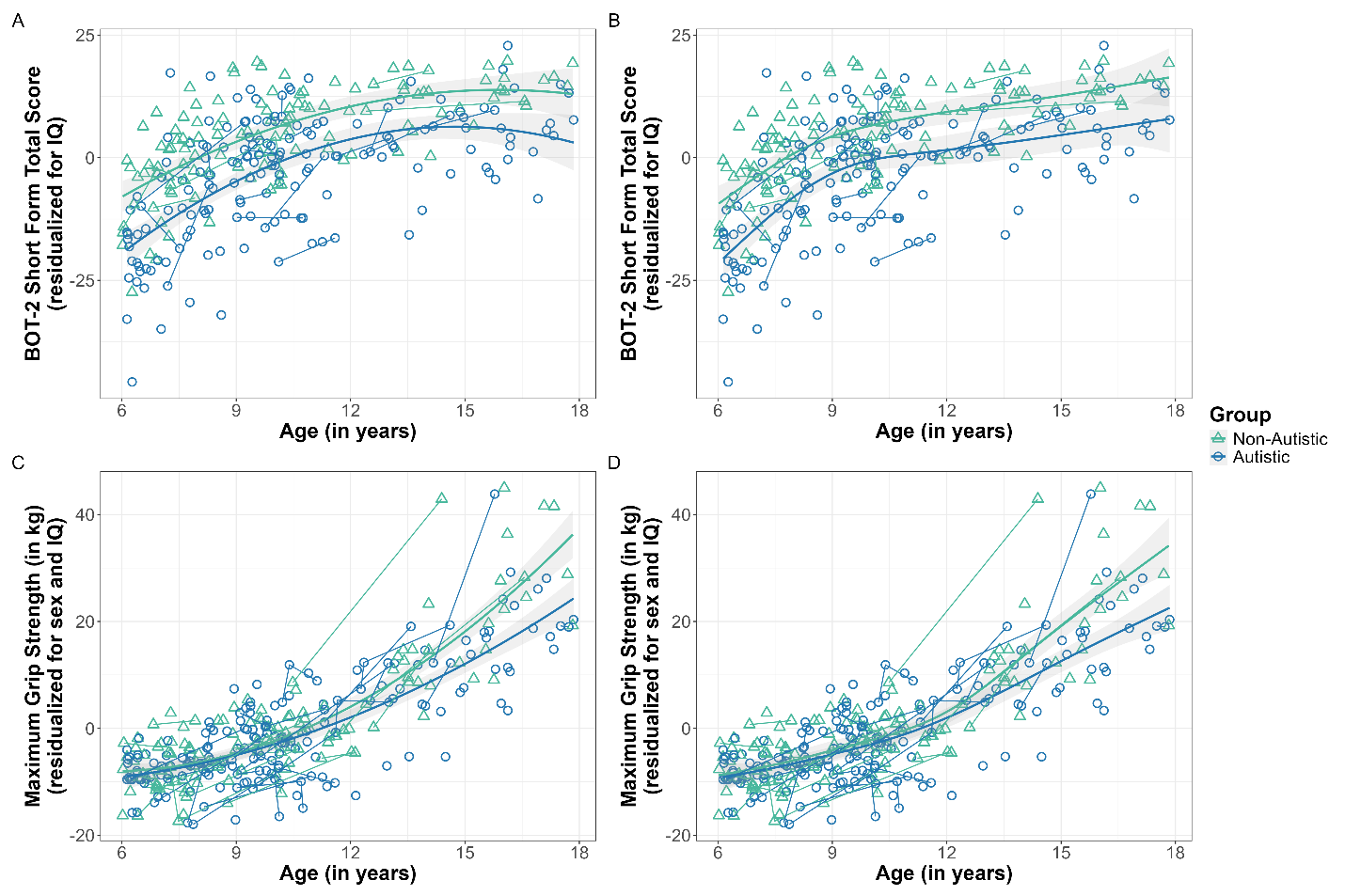


*Note*. BOT-2 SF = Bruininks-Oseretsky Test of Motor Proficiency, Second Edition, Short Form.

*Supplementary* *Figure 3.* Age-related changes on the BOT-2 SF (A) and maximal grip (B) in autistic versus non-autistic people, controlling for IQ (BOT-2 SF and grip) and sex (grip only), in identical samples of participants. Each participant in this subset had complete BOT-2 SF and grip strength data. Patterns of age-related changes in this subset of participants remain nearly identical to patterns of age-related changes in the entire sample.


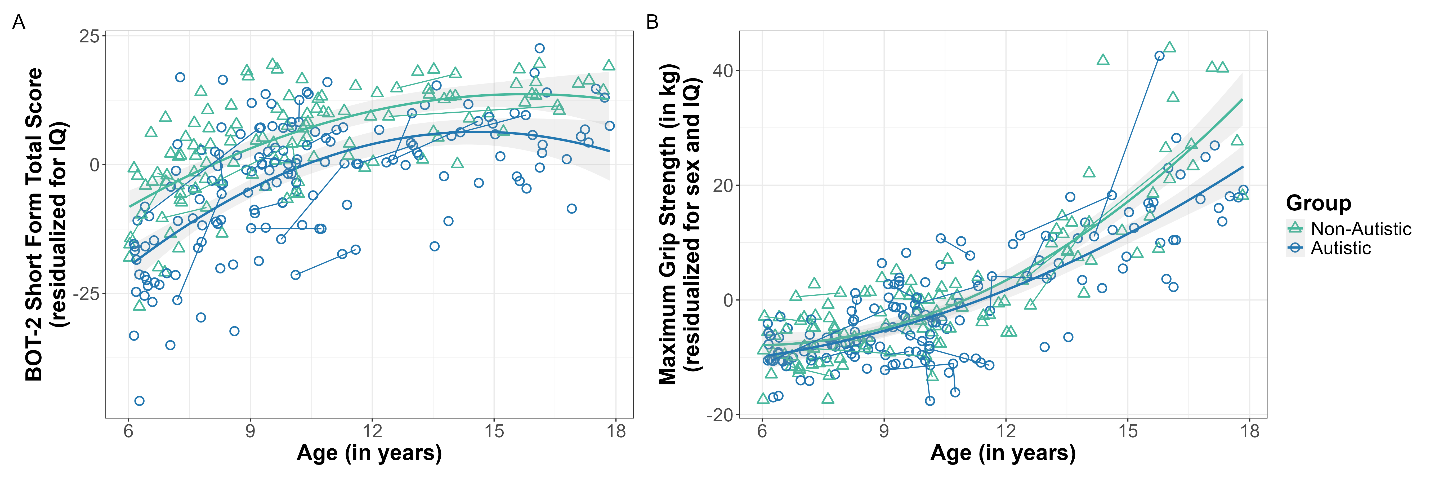


*Note*. BOT-2 SF = Bruininks-Oseretsky Test of Motor Proficiency, Second Edition, Short Form, IQ = intelligence quotient, kg = kilograms.

*Supplementary* *Figure 4.* Correlations between maximal grip strength and BOT-2 SF total point scores (A) and BOT-2 SF strength subtest average *z*-scores (B). Maximum grip strength was moderately correlated with both BOT-2 SF total point scores and BOT-2 SF average z-scores on the strength subtest. BOT-2 SF = Bruininks-Oseretsky Test of Motor Proficiency, Second Edition, Short Form.


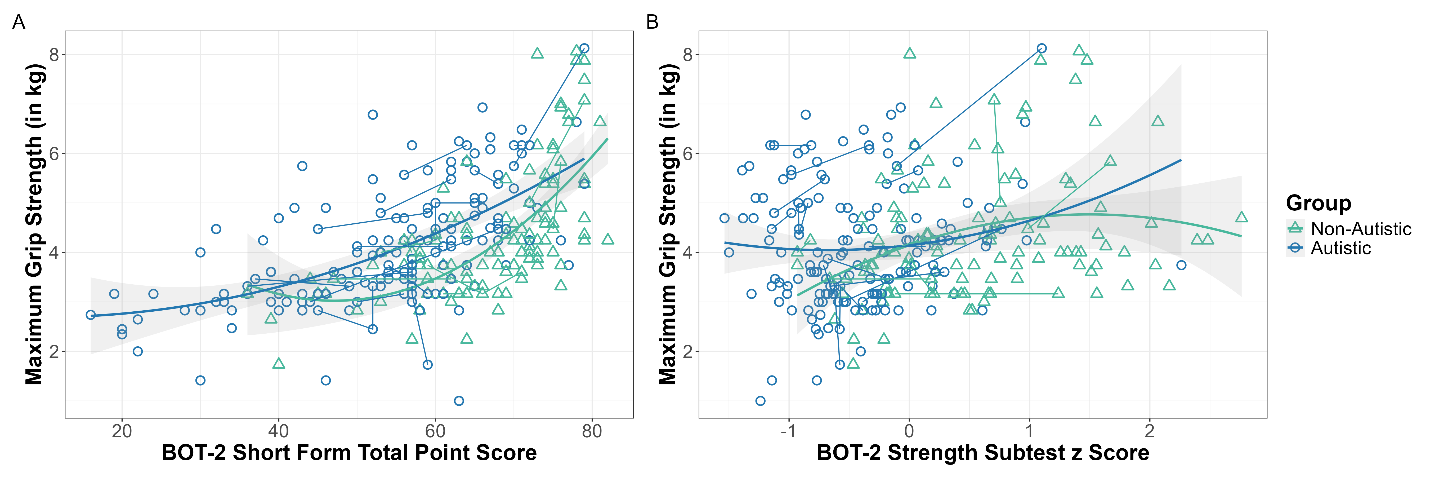


*Supplementary* *Figure 5.* Proportion of participants at ceiling and floor on each item of the BOT-2 SF by age in autistic versus non-autistic participants. The black line represents 15% cutoff utilized for classifying ceiling/floor effects. BOT-2 SF = Bruininks-Oseretsky Test of Motor Proficiency, Second Edition, Short Form


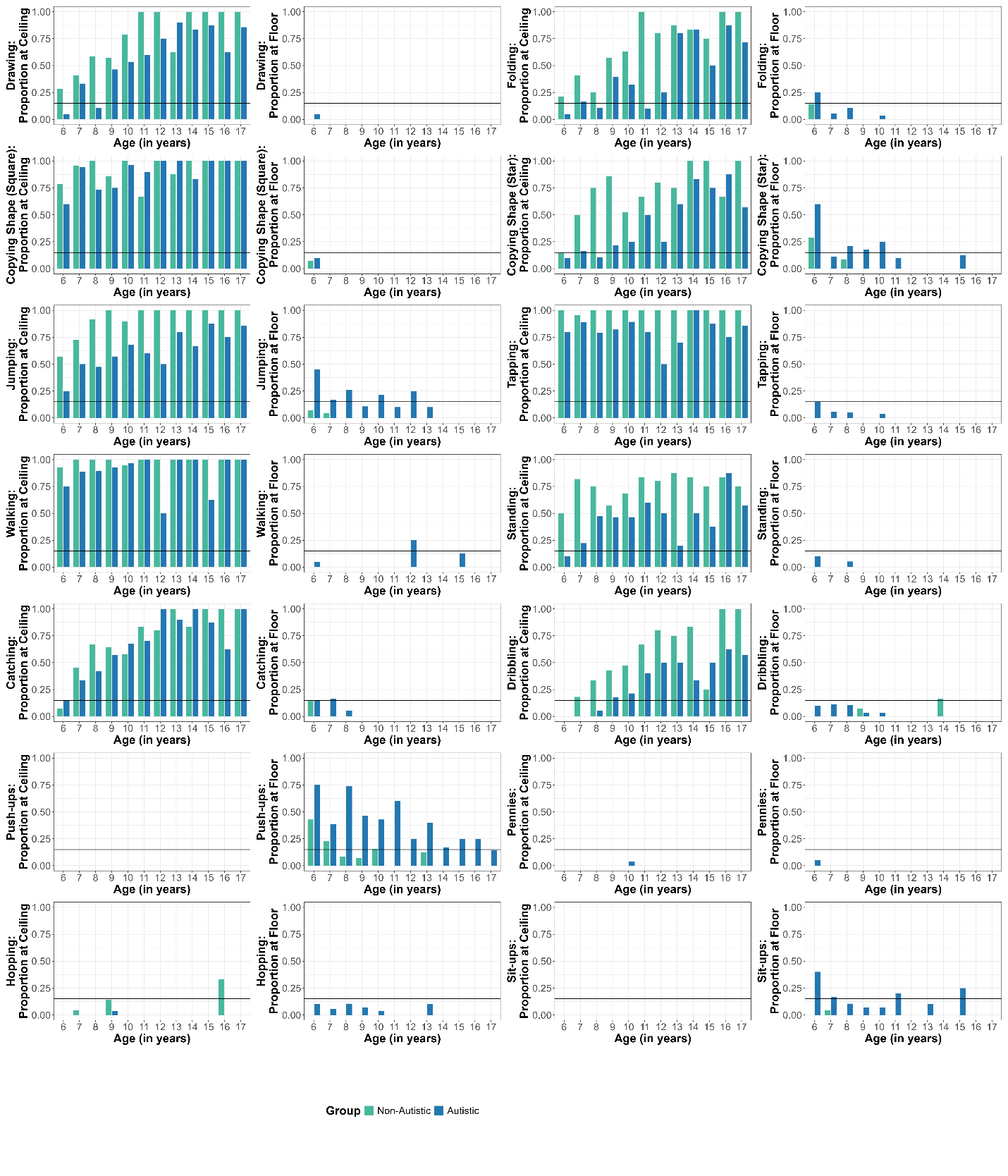

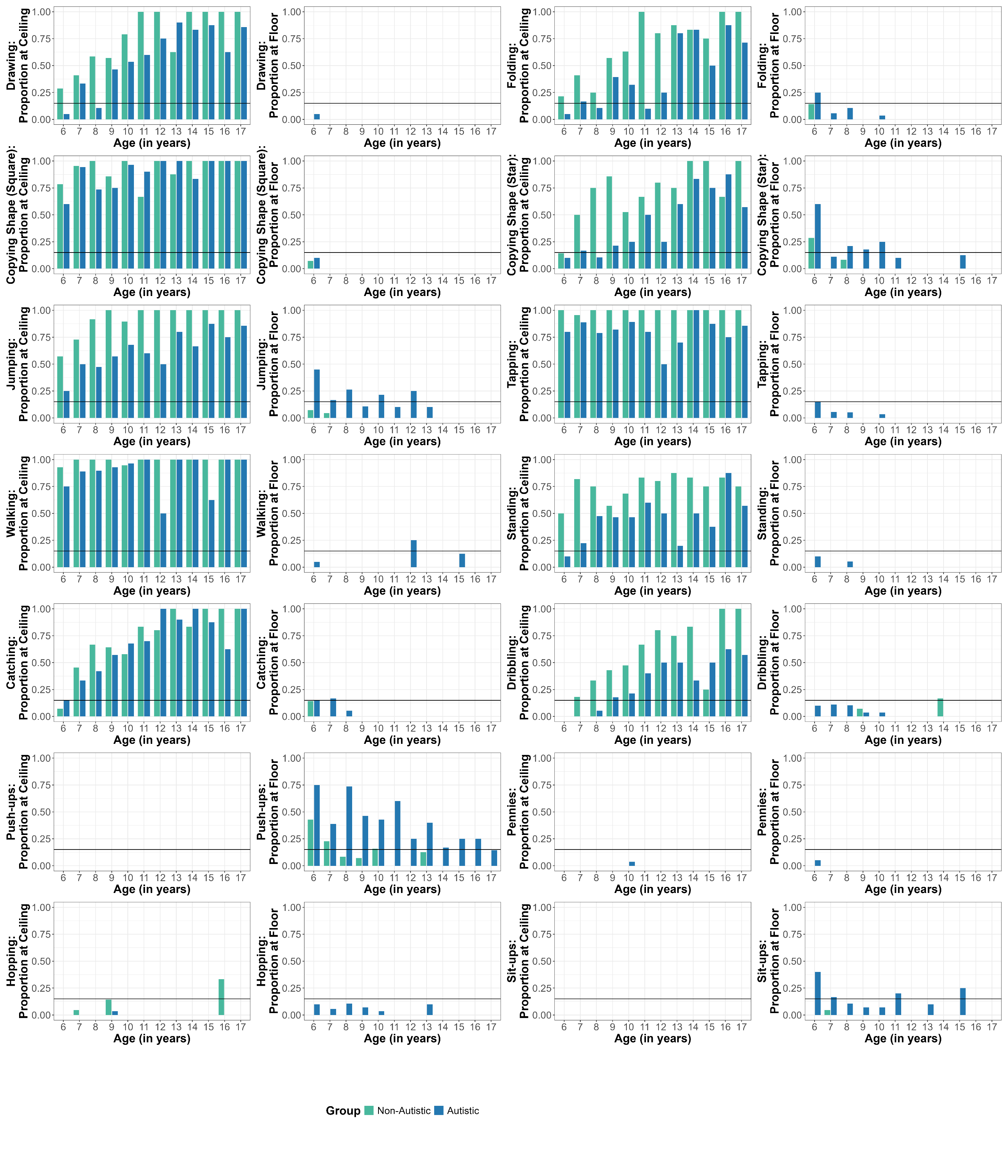

Supplement: Supplementary file 1 — Table S1: Additional inclusion/exclusion criteria that varied across studies. Table S2: Participants included in each Aim and corresponding analysis. Table S3: Results of linear mixed effects analyses of age‐related motor skills in autistic versus non‐autistic people using BOT‐2 SF standard scores over total point scores. Table S4: Results of linear mixed effects analyses of age‐related motor skills in autistic versus non‐autistic youth, in the identical samples of participants for BOT‐2 SF and grip strength. Patterns of age‐related changes in this subset of participants remain nearly identical to patterns of age‐related changes in the entire sample. Figure S1: Age‐related changes in maximal grip in autistic versus non‐autistic youth with percentile rank overlays. Panels A and B show participant grip strength overlaid with sex‐specific normative percentile lines (10th, 25th, 50th, 75th, and 90th percentiles) for males and females, respectively. BOT‐2 SF = Bruininks‐Oseretsky Test of Motor Proficiency, Second Edition, Short Form. Figure S2: Age‐related changes on the BOT‐2 SF (first row) and maximal grip (second row) in autistic versus non‐autistic youth using linear (left column) versus spline (right column) modeling. Panels A and C show linear modeling of BOT‐2 SF and maximal grip strength scores, respectively, Panels B and D show spline modeling of BOT‐2 SF and maximal grip strength scores, respectively. Because linear (with quadratic fit) and spline modeling rendered nearly identical fit lines, we opted to report the linear modeling for parsimony. Figure S3: Age‐related changes on the BOT‐2 SF (A) and maximal grip (B) in autistic versus non‐autistic people, controlling for IQ (BOT‐2 SF and grip) and sex (grip only), in identical samples of participants. Each participant in this subset had complete BOT‐2 SF and grip strength data. Patterns of age‐related changes in this subset of participants remain nearly identical to patterns of age‐related changes in the entire [file AUR-19-0-s001.docx]
